# Enhanced revision rhinoplasty with processed costal cartilage guided by preoperative computed tomography and 3D scanning

**DOI:** 10.1186/s40902-024-00422-z

**Published:** 2024-03-28

**Authors:** Pawel Szychta

**Affiliations:** 1 Dr Szychta Clinic chirurgiaplastyczna.pl, Gdansk, Poland; 2 Department of Surgical Oncology and Breast Diseases, Polish Mother’s Memorial Hospital Research Institute, Lodz, Poland

**Keywords:** Revision rhinoplasty, Costal graft integration, Precision surgery, Nasal Obstruction Symptom Evaluation, 3D scanning in rhinoplasty, Aesthetic outcomes in revision rhinoplasty, Clinical implications in rhinoplasty

## Abstract

**Background:**

Revision rhinoplasty presents unique challenges, particularly in achieving structural integrity and aesthetic harmony. This study explores the efficacy of costal grafts in addressing these challenges, focusing on anatomical corrections and patient outcomes.

**Materials and methods:**

A prospective analysis was conducted on patients undergoing revision rhinoplasty with costal grafts. An algorithmic approach was applied to tailor the surgical technique to individual anatomical needs, documented through pre- and postoperative assessments, including CT imaging and 3D scanning.

**Results:**

A total of 34 patients were included. Significant improvements were noted in nasal structure and function post-surgery. The mean NOSE score improved from 94.47 ± 5.48 preoperatively to 12.59 ± 13.43 postoperatively, and the mean ROE score increased from 18.44 ± 10.02 to 92.65 ± 13.00, indicating substantial enhancement in both nasal airway function and patient satisfaction. The use of costal grafts facilitated effective corrections for a broad spectrum of nasal deformities, with a complication rate of 2.94%.

**Conclusions:**

Costal grafts in revision rhinoplasty offer a versatile and effective solution for complex nasal deformities. The algorithmic approach used in this study enhances repeatability and outcomes, suggesting a promising avenue for achieving desired aesthetic and functional results in revision cases. Further research is warranted to optimize techniques and evaluate long-term outcomes.

**Level of evidence:**

II.

## Introduction

Revision rhinoplasty represents a multifaceted challenge in plastic surgery, particularly when addressing functional and aesthetic concerns arising from primary procedures performed elsewhere [1, 2]. Traditional revision rhinoplasty faces challenges including scar tissue formation, distorted nasal anatomy, and limited availability of native cartilage for grafting. These factors complicate surgical planning and execution, increasing the risk of suboptimal aesthetic and functional outcomes. Addressing these issues requires intricate surgical techniques, a thorough understanding of nasal structure, and, often, the use of external grafting materials. These complexities underscore the need for advancements in revision rhinoplasty approaches to improve predictability and patient satisfaction. Therefore, revision rhinoplasty remains an intricate and demanding facet of plastic surgery, often necessitating innovative approaches to address complex anatomical and aesthetic issues [3]. Patients seeking revision procedures frequently present with challenges that extend beyond the scope of primary rhinoplasty, compelling surgeons to explore novel techniques and solutions to achieve harmonious outcomes [1].

In revision rhinoplasty, costal grafts are selected for their exceptional durability and volume, addressing the challenges of insufficient nasal structure support and scar tissue from previous surgeries. These grafts, derived from rib cartilage, offer a robust material that can be carved to precise specifications, ensuring structural integrity and aesthetic goals are met. Their value lies in their ability to provide ample tissue for complex reconstructions, making them ideal for correcting significant deformities or augmentations. This choice reflects a strategic approach to overcome limitations of other graft materials, facilitating long-term success and patient satisfaction in revision procedures [4].

This study aims to unravel the complexities of indications for revision rhinoplasty with costal grafts, offering a unique lens into the preoperative planning strategies that leverage advanced imaging modalities, including computed tomography (CT) and 3D scanning. The surgical techniques employed, as well as functional and aesthetic outcomes, are systematically examined to distill insights that may guide future practices in the field. This study aims to outline the criteria for employing costal grafts in revision rhinoplasty, elucidate the intricacies of preoperative planning, and describe the surgical methodologies utilized [5]. Objectives also include quantifying functional outcomes via the Nasal Obstruction Symptom Evaluation (NOSE) scale and assessing aesthetic satisfaction through the Rhinoplasty Outcome Evaluation (ROE) questionnaire, both at initial consultation and at a 3-month postoperative follow-up [6, 7]. Additionally, this research seeks to advocate for the incorporation of precision techniques to optimize results in complex cases and to integrate the multifaceted aspects of revision surgery with the nuanced use of costal grafts, thereby enhancing patient outcomes.

## Methods

The study was conducted in accordance with ethical standards and was approved by the Ethical Committee of the Polish Mother’s Memorial Hospital — Research Institute in Lodz in Poland. Informed consent was obtained from all participants prior to their inclusion in the study. This prospective study involved patients who sought revision rhinoplasty from 2022 to 2023 for functional and aesthetic concerns following primary procedures performed in other institutions. Inclusion criteria encompassed individuals experiencing nasal obstruction and dissatisfaction with the aesthetic outcomes of their initial rhinoplasty. Patients with a history of trauma or other medical conditions affecting nasal function were excluded.

Indications for revision rhinoplasty were meticulously assessed, considering both functional impairments and aesthetic dissatisfaction. Functional issues included persistent nasal obstruction, airway obstruction, and nasal valve collapse, while aesthetic concerns encompassed asymmetry, contour irregularities, and dissatisfaction with the nasal appearance.

**Fig. 1:**
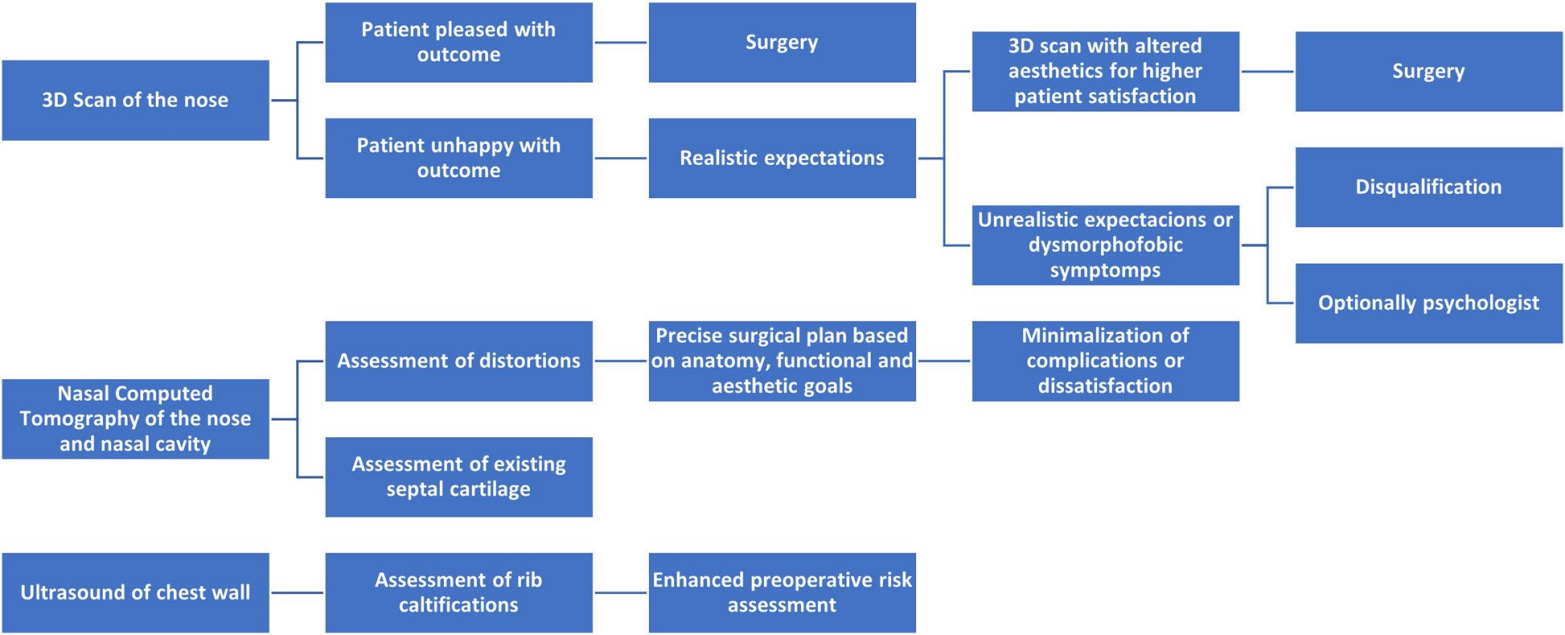
Diagrammatic representation of the decision-making process in preoperative planning for secondary rhinoplasty with processed rib cartilage using CT scans, 3D imaging, and US scan

Preoperative planning involved throughout aesthetic analysis, with depiction of the list of anatomical features to be reconstructed (Table 1). The additional tests involved advanced imaging modalities to facilitate a comprehensive understanding of each patient’s nasal anatomy. Computed tomography (CT) scans of the nose and nasal cavity, 3D scanning of the nose, and ultrasound (US) scan of chest wall were all employed to visualize the underlying structures, assess grafting needs, and guide the development of a tailored surgical plan (Fig. 1) [8, 9]. The information gathered from these imaging techniques informed decisions regarding costal graft selection, dimensions, and placement.

**Table 1:** Desired characteristics of nasal skeleton anatomy, required grafts, and maneuvers for algorithmic approach in revision rhinoplasty for enhanced repeatability and outcomes

Anatomical characteristic	Detailed description of ideal characteristics	Required grafts	Specific maneuvers for achieving ideal characteristics
**1. Nasal dorsal contour**	• Smooth, continuous dorsal contour without irregularities • Adequate height and projection with a natural slope from radix to tip	Spreader graft, onlay graft	o Precise sculpting of spreader grafts for maintaining dorsal width and enhancing projection • Application of onlay graft for smooth contour and augmentation
**2. Nasal bridge symmetry**	o Bilateral symmetry along the nasal bridge • Even width and alignment of bony and cartilaginous components for balanced appearance	Spreader graft, lateral crural graft	o Symmetrical placement of spreader grafts for maintaining bridge width • Application of lateral crural grafts for balance and alignment
**3. Septal integrity and alignment**	o Straight and well-aligned nasal septum • Preservation of septal support to ensure proper structural foundation	Septal extension graft	o Utilization of septal extension graft for straightening and aligning the nasal septum • Preservation and reinforcement of septal support during surgery
**4. Nasal tip definition**	o Well-defined tip with appropriate projection • Symmetrical and proportional tip contour contributing to overall facial harmony	Lower lateral cartilage graft (LLCG)	o Sculpting of LLCG for tip projection and contouring • Precise placement for symmetrical and proportional tip definition
**5. Alar rim symmetry and positioning**	o Symmetric alar rims with consistent positioning • Proper alignment with the midline for balanced nostril shapes	Alar rim graft	o Application of alar rim grafts for achieving symmetry and proper positioning • Real-time adjustments for balanced nostril shapes
**6. Adequate internal valve function**	o Patent internal nasal valves for unobstructed airflow • Preservation or restoration of internal nasal valves to prevent functional issues	Spreader graft, septal extension graft	o Placement of spreader grafts for maintaining internal valve patency • Utilization of septal extension grafts to reinforce and support internal valves
**7. Nasal base width proportionality**	o Harmonious nasal base width in relation to other facial features • Avoidance of excessive flaring or constriction	Alar base reduction graft, columellar strut	o Utilization of alar base reduction grafts for proportional width • Application of a columellar strut for support and proportional base width
**8. Smooth transition zones**	o Seamless transitions between dorsal contour, sidewalls, and tip • The absence of abrupt changes for a pleasing overall nasal appearance	Spreader Graft, Onlay Graft	o Incorporation of spreader grafts to ensure smooth transitions between dorsal and sidewalls • Application of onlay grafts for gradual and natural contour changes
**9. Adequate columellar show**	o Appropriate columellar show contributing to balanced nasal proportions • Avoidance of excessive or insufficient columellar visibility	Columellar strut	o Placement of a columellar strut for controlled and balanced columellar show • Real-time adjustments for the desired aesthetic proportions
**10. Nostril shape and size harmony**	o Well-defined and symmetric nostril shapes • Proportional nostril size in relation to overall nasal structure and facial aesthetics	Alar rim graft	o Application of alar rim grafts for shaping and defining nostrils symmetrically • Real-time adjustments to ensure proportional nostril size and shape
**11. Satisfactory nasolabial angle**	o Optimal nasolabial angle for a natural aesthetic appearance • Avoidance of excessive upward or downward rotation for facial harmony	Septal extension graft	o Utilization of septal extension grafts for precise control over the nasolabial angle • Dynamic assessment for refinement during surgery
**12. Consistent nasal tip rotation**	o Consistent and balanced tip rotation on both sides • Avoidance of tip asymmetry and unnaturally high or low tip positioning	Lower lateral cartilage graft (LLCG)	o Sculpting of LLCG for achieving consistent tip rotation • Real-time adjustments to ensure symmetrical and balanced tip positioning

**Fig. 2:**
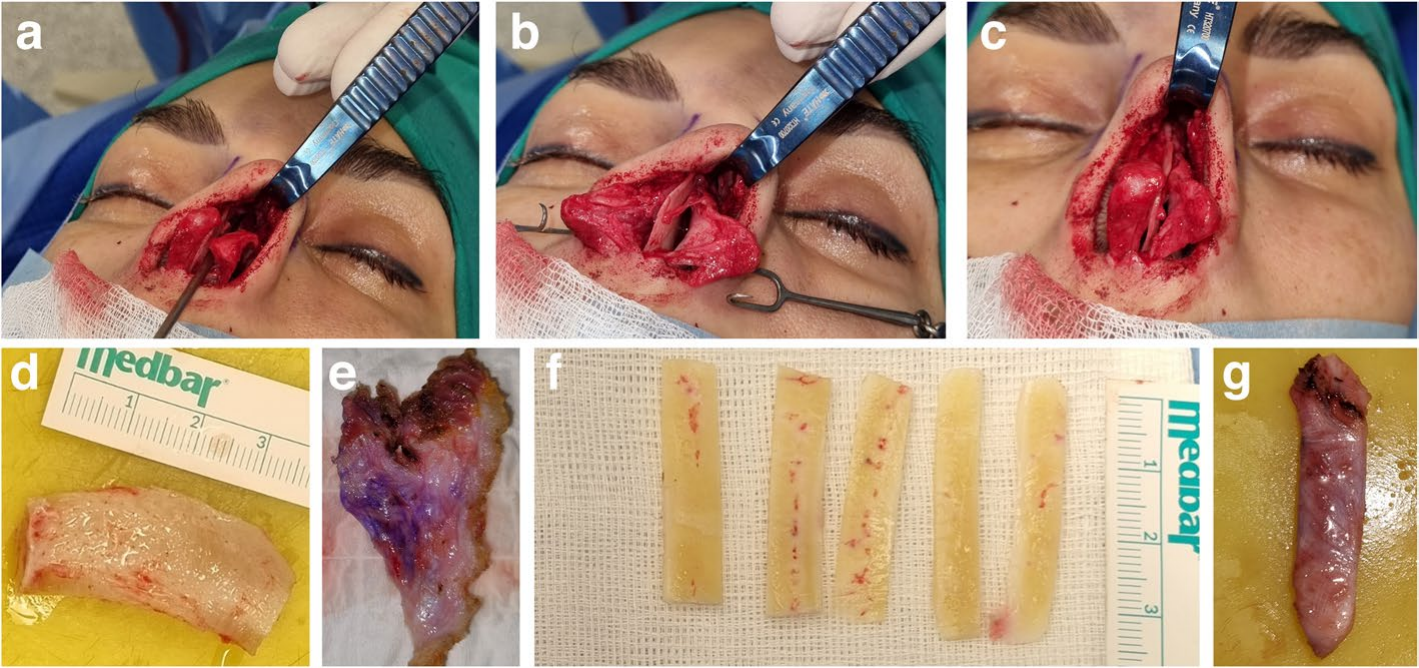
Intraoperative photographs of the patient A. **a** Distortions of the lower lateral cartilages, seen in preoperative CT, which in turn enabled tailored planning of the size and shapes of the required rib grafts. **b** Septal deviation. **c** Collapse of the upper lateral cartilages with very narrow internal nasal valves, together with irregularities of the septum in its dorsal edge. **d** Rib graft immediately after harvest. **e** Deep fascia graft. **f** Processed rib grafts, guided by preoperative CT-scanning. **g** Onlay rib graft wrapped up by deep fascia for enhanced contouring

All surgeries were performed by the author focused in rhinoplasty. A combination of autologous costal cartilage grafts and standard rhinoplasty techniques was employed (Table 1, Fig. 2). Achieving optimal outcomes in revision rhinoplasty demands an innovative surgical approach, particularly when incorporating costal grafts. Therefore, for standardization, the comprehensive treatment protocol was designed for the study. The detailed protocol for the desired outcome based on checklist of the preferred postoperative anatomical characteristics listed in Table 1 reflects the culmination of our experience in enhancing revision rhinoplasty perspectives through precision with costal graft integration. Precision harvesting of costal grafts involves small incision along the chest wall with choice of rib (sixth or seventh) based on graft size and anatomy. Artistic sculpting defines grafts treated as sculptural elements. Layered sculpting is undertaken for three-dimensional structural support and aesthetic harmony. Layered graft placement technique is defined by strategic layering for foundational support and contour refinement. Precision is essential in graft placement for correction of functional and aesthetic issues. Dynamic intraoperative adjustments are usually required for real-time assessment of nasal dynamics. Real-time adjustments are based on evolving nasal structure for optimal graft placement. Seamless integration with nasal framework is feasible by meticulous positioning for seamless integration. Contouring grafts can be utilized to match surrounding nasal structures for natural and proportionate appearance. The surgical approach was tailored to address each patient’s unique anatomical challenges, focusing on restoring structural integrity, improving nasal function, and achieving aesthetically pleasing outcomes. The harvested rib cartilage, informed by these imaging modalities, is sculpted into various graft types, each addressing specific clinical scenarios:

- Dorsal onlay grafts: These grafts, enhanced by CT scan insights, are used for augmenting the nasal dorsum, especially in cases of saddle nose deformity. The CT scan’s cross-sectional view aids in determining the precise height and contour of the graft required.
- Spreader grafts: Informed by both 3D and CT scans, spreader grafts are used to widen a narrow middle vault or correct internal valve insufficiency. The 3D scan helps in gauging the external width, while the CT scan provides details about the internal nasal valve angle and septal alignment.
- Columellar strut grafts: Primarily enhanced by 3D scanning, these grafts support the nasal tip and prevent its collapse, particularly critical in revision cases where prior surgery may have weakened the tip support.
- Tip grafts: Refined by the external details from the 3D scan, these grafts are used for tip refinement and projection, allowing for precise aesthetic enhancements in line with the patient’s desired outcome.
- Alar batten or rim grafts: CT scans are particularly useful in planning these grafts, used to correct alar retraction or collapse, as they provide a clear view of the lateral nasal wall and alar cartilage status.
- L-strut grafts: These provide major structural support and are planned based on a comprehensive analysis from both scans, ensuring the grafts are strong enough to sustain the nasal framework, especially in cases of major septal compromise or previous over-resection.

Each graft type is meticulously crafted based on the specific dimensions and characteristics identified in the preoperative scans. The precision in graft preparation and placement, guided by these advanced imaging techniques, is crucial in addressing the unique challenges of revision rhinoplasty. The 3D scan’s ability to predict the external appearance postoperatively and the CT scan’s capacity to reveal the internal nasal structure collectively ensure that the grafts are not only functionally effective but also aesthetically congruent with the patient’s facial anatomy.

Functional outcomes were quantified using the Nasal Obstruction Symptom Evaluation (NOSE) scale ranging from 0 to 100, administered to participants at baseline and 3 months postoperatively [6]. The NOSE scale provided a quantitative assessment of improvements in nasal obstruction symptoms. Additionally, patient aesthetic satisfaction was evaluated using the Rhinoplasty Outcome Evaluation (ROE) questionnaire at the same intervals, offering insights into subjective perceptions of the aesthetic outcomes [7].

Descriptive statistics were used to summarize patient demographics and clinical characteristics. Paired *t*-tests were employed to analyze pre- and postoperative NOSE scale scores, assessing the significance of functional improvements. Aesthetic satisfaction scores from the ROE questionnaire were similarly analyzed.

Concluding clinical implications drawn from the study were based on the collective experiences of the series, aiming to provide valuable insights for other plastic surgeons engaged in revision rhinoplasty. These implications encompassed considerations for preoperative planning, surgical techniques, and expectations for functional and aesthetic outcomes.

## Results

**Fig. 3:**
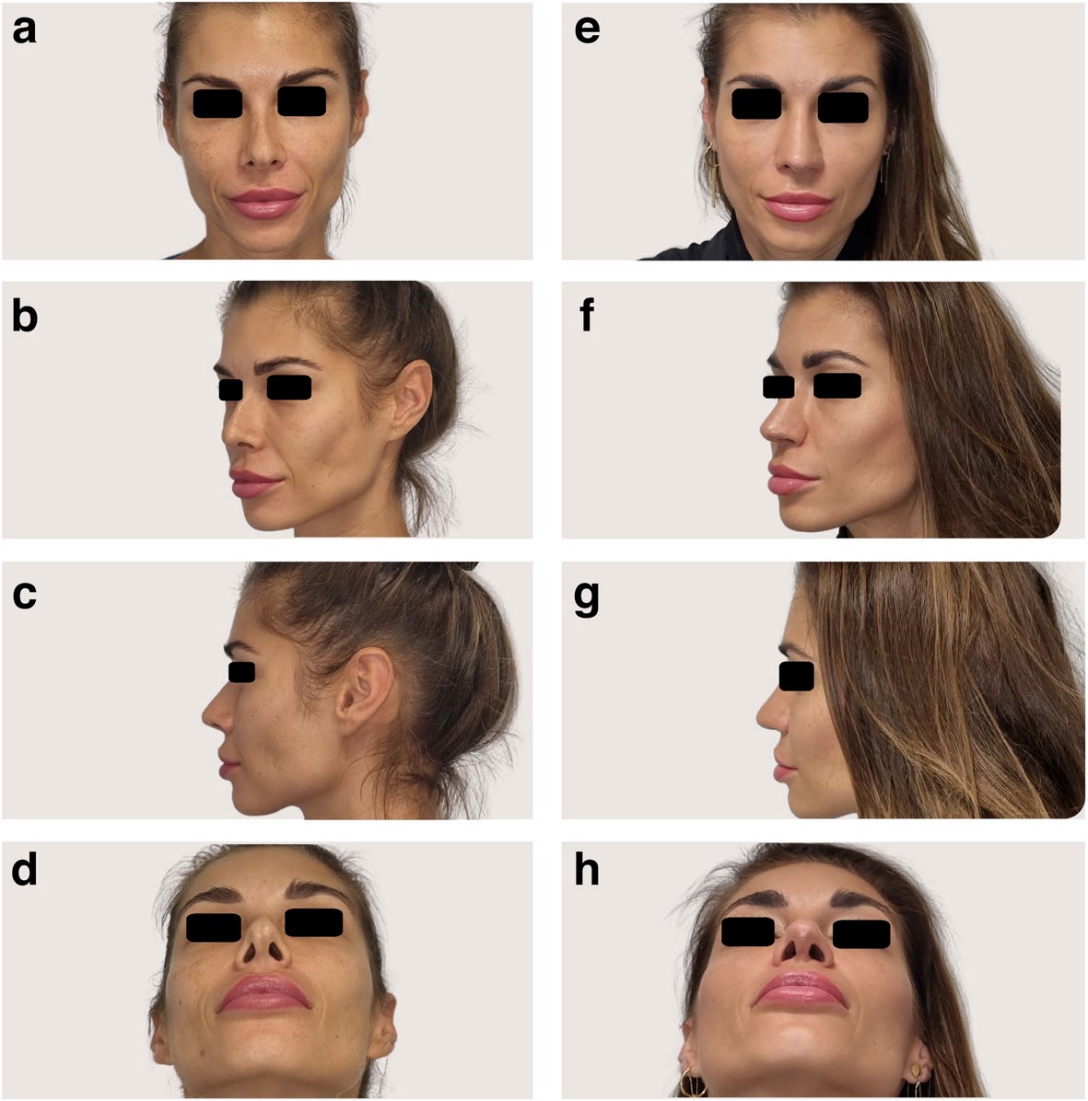
CT-assisted and 3D-assisted revision rhinoplasty with costal graft in patient A with severe functional and aesthetic issues after previous rhinoplasty. **a**–**d** Pre- and **e**–**h** postoperative photos

**Fig. 4:**
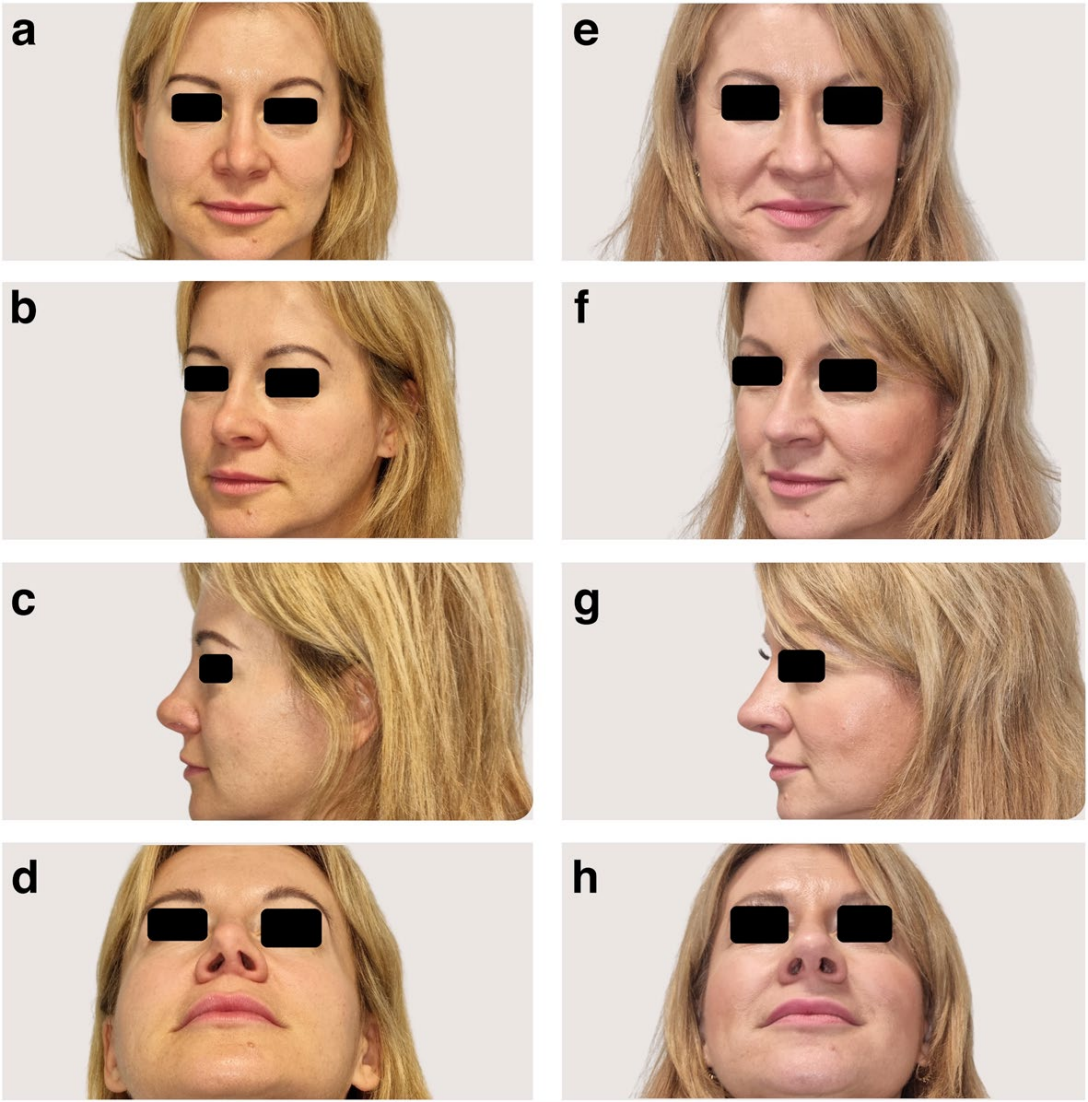
CT-assisted and 3D-assisted revision rhinoplasty with costal graft in patient B with severe functional and aesthetic issues after previous rhinoplasty. **a**–**d** Pre- and **e**–**h** postoperative photos

A series of 34 patients, aged 26–56 years old (67.65% women, 32.35% men), underwent revision rhinoplasty with costal graft integration in response to functional and aesthetic concerns stemming from primary rhinoplasties performed in other institutions (Table 2). Indications for revision surgery were diverse, reflecting a spectrum of functional and aesthetic challenges. Nasal airway obstruction (100%) and dissatisfaction with nasal appearance (91.43%) were predominant indications, highlighting the complex nature of cases (Figs. 3 and 4).

**Table 2:** Comparative statistical evaluation of pre- and post-rhinoplasty patient outcomes and characteristics

Parameter	Before/after rhinoplasty	Median	SD	Mean	Min	Max	Statistical significance
Age		38	8.76	40.29	26	56	-
NOSE	Before	96	5.48	94.47	84	100	*p* < 0.05
	After	12	13.43	12.59	0	52	
ROE	Before	18.5	10.02	18.44	0	37	*p* < 0.05
	After	100	13.00	92.65	60	100	

*SD* standard deviation

**Fig. 5:**
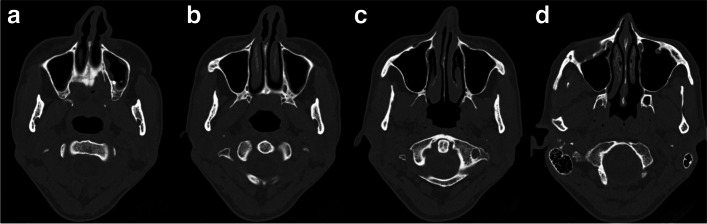
CT of the external nose and nasal cavity of the patient A, depicting **a** asymmetric tip, narrow right external valve with weak left external valve, thickened soft tissues of septum, and inwards rotated lateral crus of lower lateral cartilage bilaterally; **b** hypertrophic lower turbinates, septal deviation, and adhesion between septum and right lower turbinate; **c** collapsed internal nasal valves and subtle septal spur; and **d** inwards rotated nasal bone following too high osteotomy, wide high septum, and concha bullosa bilaterally

**Fig. 6:**
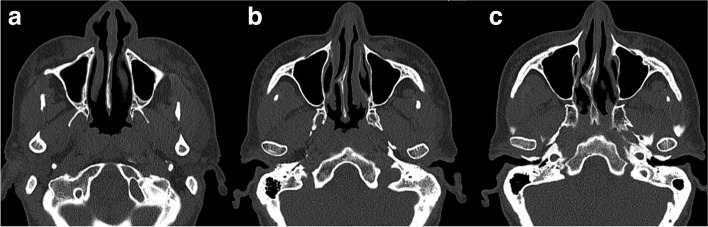
CT of the external nose and nasal cavity of the patient B, depicting the following: **a** thickened and irregular mucosal lining of septum; **b** collapsed internal valves bilaterally, septal spur with septal deviation, and medialized left lower turbinate; **c** large septal spur located posteriorly, asymmetric short nasal bones, right pointed inwards, left pointed more perpendicularly, and asymmetry of surrounding facial features

Utilizing computed tomography (CT) and 3D scanning, preoperative planning facilitated a meticulous assessment of nasal anatomy (Table 3). Imaging revealed varying degrees of anatomical irregularities, guiding the selection of autologous costal grafts and influencing significantly the surgical strategy (Figs. 2, 5 and 6).

**Table 3:** Anatomical distortions of nasal anatomy detectable in CT, specific maneuvers for correction, and the corresponding specific grafts used for correction

Anatomical distortion detectable in CT	Maneuvers for correction	Graft types for correction
**Nasal valve collapse**	Restore and maintain nasal valve angle	Spreader graft, extended spreader graft
**Alar collapse**	Enhance alar support	Alar batten graft
**Columellar retraction**	Improve columellar projection	Columellar strut graft
**Weak tip projection**	Enhance and define nasal tip projection	Tip graft
**Lateral crus weakness**	Reinforce lateral crus support	Lateral crural strut graft
**Internal nasal valve collapse**	Stabilize internal nasal valve	Spreader graft, extended spreader graft
**Nasal dorsum irregularities**	Smooth nasal dorsum contour	Onlay graft
**Tip irregularities**	Refine nasal lip shape	Shield graft
**Overall nasal aesthetic enhancement**	Achieve harmonious nasal proportions	Cap graft
**Nasal dorsum profile augmentation**	Add volume to nasal dorsum	Onlay graft
**Nasal tip aesthetic refinement**	Sculpt and refine nasal tip appearance	Shield graft, cap graft

The surgical approach involved the strategic integration of autologous costal cartilage grafts to address anatomical deficiencies. Grafts were meticulously placed to reconstruct nasal structures, providing durable support and contour refinement. Standard rhinoplasty techniques were seamlessly incorporated to achieve a harmonious blend of function and aesthetics.

Functional improvements were quantified using the Nasal Obstruction Symptom Evaluation (NOSE) scale (Table 2). Baseline NOSE scores averaged 94.47 (out of maximum 100), demonstrating a significant reduction to 12.59 (*p* < 0.05) at the 3-month postoperative assessment. The outcomes indicated a tangible enhancement in nasal function, alleviating obstructive symptoms. Evaluation of patient aesthetic satisfaction utilized the Rhinoplasty Outcome Evaluation (ROE) questionnaire at baseline and 3 months postoperatively. The ROE scores demonstrated a significant improvement of 74.21 points (*p* < 0.05), reflecting enhanced patient contentment with the aesthetic outcomes of revision rhinoplasty.

During the follow-up, only one complication was encountered in form of infection managed with antibiotics, highlighting the importance of careful patient selection, meticulous surgical execution, and comprehensive postoperative care.

Concluding clinical implications drew from the collective experiences of the study. Noteworthy considerations encompassed the efficacy of costal graft integration, refined preoperative planning strategies, and the potential for transformative outcomes in revision rhinoplasty. These implications seek to inform and guide fellow plastic surgeons facing similar challenges in their practice (Table 4).

**Table 4:** Comprehensive outline of the clinical implications drawn from the study

Clinical implications	Clinical problems addressed	Surgical techniques	Applicability of CT and 3D scanning
**Correction of nasal valve collapse**	Impaired nasal airflow due to valve collapse	Spreader graft placement to reinforce the nasal valve	CT aids in precise evaluation of nasal valve anatomy for targeted correction
**Alar support enhancement**	Insufficient alar support causing deformities	Alar batten grafts for enhanced lateral support	3D scanning for detailed assessment of alar support and graft placement planning
**Improved columellar projection**	Poor columellar projection affecting aesthetics	Columellar strut grafts for enhanced projection	CT imaging for insights into columellar anatomy and surgical planning
**Nasal tip projection refinement**	Suboptimal tip projection leading to imbalance	Tip grafts for precise tip projection refinement	3D scanning for precise measurement and planning for optimal tip projection
**Reinforcement of lateral crus support**	Weak lateral crus support causing nasal deformities	Lateral crural strut grafts for lateral support	CT scans for understanding lateral crus dynamics and support reinforcement
**Stabilization of internal nasal valve**	Internal nasal valve collapse affecting breathing	Onlay grafts to stabilize the internal nasal valve	CT imaging aids in identifying internal nasal valve issues and guides graft placement
**Smooth nasal dorsum contour**	Dorsal irregularities impacting nasal aesthetics	Onlay grafts for dorsal contour refinement	3D scanning assists in evaluating dorsal irregularities for precise contouring
**Refinement of nasal tip shape**	Undesirable tip shape detracting from overall appearance	Tip grafts for reshaping and refining the nasal tip	CT scans guide reshaping of the nasal tip for aesthetic refinement
**Achieving harmonious nasal proportions**	Nasal disproportions impacting overall facial harmony	Combination graft techniques for proportional balance	3D scanning aids in visualizing overall nasal proportions and guides surgical planning
**Nasal dorsum volume augmentation**	Insufficient nasal dorsum volume affecting aesthetics	Onlay grafts for dorsal volume augmentation	CT scans contribute to volume assessment and guide graft selection for augmentation
**Sculpting and refining nasal tip appearance**	Nasal tip irregularities affecting aesthetic appeal	Precision sculpting and tip refinement techniques	3D scanning offers a detailed view for sculpting and refining the nasal tip
**Comprehensive correction of nasal deformities**	Multifaceted nasal deformities post primary rhinoplasty	Combined grafting and sculpting techniques	Integrated use of CT and 3D scanning ensures a holistic approach to correction

## Discussion

The findings of this prospective study shed light on the intricate landscape of revision rhinoplasty, specifically when augmented by the precision associated with costal graft integration [10]. The predominant indications for revision surgery, including nasal obstruction and dissatisfaction with nasal appearance, underscore the complexity of cases encountered in this series [11]. The quantifiable improvements in functional outcomes, as measured by the Nasal Obstruction Symptom Evaluation (NOSE) scale, reflect the tangible success of revision rhinoplasty with costal grafts. The significant reduction in NOSE scores at 3 months postoperatively emphasizes the efficacy of the surgical interventions in alleviating nasal obstruction symptoms. Simultaneously, the enhanced aesthetic satisfaction, as captured by the Rhinoplasty Outcome Evaluation (ROE) questionnaire, reinforces the transformative impact on patients’ subjective experiences of their nasal appearance.

The study’s outcomes align with and contribute to the evolving literature on revision rhinoplasty [12]. The use of costal grafts has been previously associated with improved structural support and durability, corroborating existing evidence in the field [13, 14]. The observed complications underscore the need for a nuanced approach, emphasizing the importance of careful patient selection and comprehensive postoperative care to mitigate adverse events [15].

The integration of 3D scanning and CT imaging in preoperative assessment facilitates a nuanced understanding of the nasal architecture, crucial for tailoring the revision surgery to individual needs [8, 9]. The 3D scan, offering a high-resolution external view, is instrumental in assessing aesthetic deformities and asymmetries. It aids in envisioning the expected outcomes and precisely planning the external modifications of the grafts, such as their shape and contour, to harmonize with the patient’s facial features (Table 3). The preoperative nasal CT serves as a crucial tool in meticulously planning and executing this intricate operation [8]. Through a comprehensive evaluation of the nasal anatomy and any prior surgical alterations, the CT scan provides invaluable insights into the patient’s nasal framework, including the presence of septal deviation, nasal bone morphology, and the integrity of existing cartilage structures [4]. This detailed assessment allows for a tailored surgical approach, particularly in the harvesting and sculpting of rib cartilage grafts [5]. By precisely delineating the dimensions and contours of the rib cartilage, the preoperative CT scan facilitates strategic planning to ensure optimal graft size and shape required for structural support and functional restoration, thereby minimizing donor site morbidity and enhancing the aesthetic and functional outcomes of the revision rhinoplasty. Furthermore, the CT evaluation aids in identifying potential challenges such as previous scar tissue, septal perforations, or asymmetric nasal anatomy, enabling the surgeon to anticipate and address these complexities during the surgical procedure. In essence, the integration of preoperative nasal CT imaging into the surgical workflow serves as a fundamental step in achieving successful outcomes in revision rhinoplasty with rib cartilage grafting, allowing for meticulous planning and precise execution tailored to the individual patient’s anatomical nuances and surgical goals.

The utility of preoperative 3D scanning in revision rhinoplasty with costal grafts represents a significant advancement in surgical planning and outcomes [9]. This technology enables precise anatomical assessment, allowing surgeons to visualize the complex structures of the nose with unparalleled clarity. By creating a detailed three-dimensional model of the patient’s nasal anatomy, surgeons can accurately plan the size, shape, and placement of costal grafts before the procedure. This preoperative insight facilitates tailored interventions, minimizes intraoperative guesswork, and enhances the accuracy of graft fitting, leading to improved aesthetic and functional results. The integration of 3D scanning technology into revision rhinoplasty with costal grafts thus marks a pivotal shift towards more predictive and patient-specific surgical outcomes.

In summary, the detailed preliminary evaluation using 3D and CT scans is pivotal in guiding the harvesting and sculpting of rib cartilage for revision rhinoplasty. This approach allows for a highly customized surgical plan, addressing a range of clinical scenarios with appropriately designed chondral grafts, thereby enhancing both the functional and aesthetic outcomes of the procedure.

Our study introduces several advancements and refinements in both the surgical technique and evaluation metrics, distinguishing it from traditional methodologies. Firstly, our approach integrates advanced imaging techniques, specifically high-resolution 3D scanning and CT imaging, not as mere diagnostic tools but as integral components of surgical planning. This goes beyond the conventional use of these imaging methods. The detailed visualization afforded by these scans allows for a more precise and tailored approach to graft harvesting and sculpting. Unlike traditional techniques, which often rely on surgeon’s experience and intraoperative assessment, our method uses these scans to create a detailed map of the nasal architecture, guiding the surgeon in crafting grafts with specific dimensions and shapes suited to each patient’s unique anatomical requirements. This precision in graft preparation, especially in the challenging realm of revision rhinoplasty, potentially reduces the risk of over- or under-correction and enhances both aesthetic and functional outcomes. Additionally, our study offers new insights into the relationship between preoperative imaging findings and postoperative outcomes. By correlating specific anatomical features identified in preoperative scans with postoperative NOSE and ROE scores, we aim to establish a more predictive model of patient outcomes. This model could potentially guide surgeons in making more informed decisions during both the planning and execution phases of the surgery.

The summarized clinical implications drawn from the current experiences serve as a guidepost for plastic surgeons navigating the challenges of revision rhinoplasty. Notably, the study advocates for the strategic integration of costal grafts, refined preoperative planning methodologies, and a comprehensive understanding of patient expectations. These implications offer insights that extend beyond the confines of the study, providing a foundation for fellow plastic surgeons seeking to enhance their practice in revisionary settings. The study presents innovative elements by integrating advanced imaging techniques (3D scanning and CT imaging) for precise preoperative planning in revision rhinoplasty with costal grafts, moving beyond traditional diagnostic uses. This approach allows for detailed mapping of nasal architecture, enabling the creation of grafts tailored to individual anatomical needs. It offers a refined surgical technique that potentially reduces the risk of over- or under-correction, enhancing both aesthetic and functional outcomes. The study also proposes a predictive model correlating preoperative imaging findings with postoperative outcomes, aiming to guide surgeons in making more informed decisions. This novel integration of technology and technique provides new insights into optimizing revision rhinoplasty outcomes.

Despite the valuable insights garnered, this study has inherent limitations. The single-center design and the absence of a control group limit the generalizability of findings. Further, the relatively short follow-up period necessitates cautious interpretation of long-term outcomes. Future research could explore larger multicenter studies with extended follow-up periods to enhance the robustness of findings and address potential confounders.

## Conclusions

In conclusion, this prospective study presents a compelling narrative of enhancing revision rhinoplasty perspectives through the integration of precision with costal grafts. The findings underscore the transformative potential of this surgical approach, offering both functional relief and enhanced aesthetic satisfaction. The clinical implications drawn from this study provide valuable insights for plastic surgeons, encouraging a nuanced and patient-centered approach to revisionary challenges. As the field continues to evolve, the lessons learned from this series contribute to the ongoing dialogue aimed at optimizing outcomes in revision rhinoplasty.

## Data Availability

Data and material are available upon reasonable request.

## Abbreviations

CT: Computed tomography
NOSE: Nasal Obstruction Symptom Evaluation scale
ROE: Rhinoplasty Outcome Evaluation questionnaire
